# Reproductive characteristics of the giant gurami sago strain ( *Osphronemus goramy* Lacepède, 1801): basic knowledge for a future hatchery development strategy

**DOI:** 10.12688/f1000research.53760.1

**Published:** 2021-09-15

**Authors:** Azrita Azrita, Hafrijal Syandri, Netti Aryani

**Affiliations:** 1 Department of Biology Education, Faculty of Education, Universitas Bung Hatta, Padang, West Sumatera, 25133, Indonesia; 2 Department of Aquaculture, Faculty of Fisheries and Marine Science, Universitas Bung Hatta, Padang, West Sumatera, 25133, Indonesia; 3 Department of Aquaculture, Faculty of Fisheries and Marine Science, Universitas Riau, Pekanbaru, Riau, 28293, Indonesia

**Keywords:** Aquaculture, giant gourami, broodfish, egg, sperm, hatchery performance.

## Abstract

**Background:** The giant gourami sago strain ( *Osphronemus goramy* Lacepède) has been approved in 2018 as a candidate for freshwater aquaculture in Indonesia. However, information on the species’ reproduction is minimal. This study analyzed the reproductive characteristics of the gourami sago strain broodfish to provide basic knowledge for a future hatchery development strategy.

**Methods:** A total of 10 female and 10 male mature gourami sago strain broodfish were measured for body weight and length, and were evaluated for their reproductive characteristics.  Breeding fish were spawned naturally in a 2×1×0.6 m concrete pond with a male-female sex ratio of 1:1. Egg weight and diameter were measured in 25 eggs per female using, respectively, ACIS AD- 600i scales with 0.01 g accuracy, and a microscope (Labo model L-711) using Canon Digital Camera Software 3 . Semen was collected using plastic syringes in 3 mL aliquots, then placed in an insulated ice-cooled container, and analyzed within two hours of collection.

**Results:** Average weights of female and male broodfish before spawning were 2180±159.78 g and 3060±134.99 g, respectively. The relative fecundity and egg diameter were 1029±36 eggs kg ^-1^ and 2.42±0.05 mm, respectively. The hatching rate and embryo survival to an eyed-egg stage were respectively 76.40±2.27% and 94.76±0.42%.  Sperm characteristics showed that volume was 0.60±0.12 ml kg ^-1^ and percentage of motile sperm was 70.04±2.27%. Female broodfish weight after spawning was strongly correlated with the weight before spawned ( *r* ^2^ = 0.999) and absolute fecundity was also strongly correlated with female broodfish weight before spawning ( *r* ^2^= 0.921). Sperm concentration was moderately correlated with sperm motility ( *r* ^2^ = 0.556) and duration of sperm motility ( *r* ^2^ = 0.502).

**Conclusions: **The gourami sago strain broodfish has suitable reproductive characteristics for the development of hatcheries. Successful natural spawning should be followed by larval weaning and feeding technology to increase growth and survival.

## Introduction

Freshwater aquaculture practiced in inland waters such as lakes, rivers, reservoirs, floodplains, oxbow lakes, and freshwater ponds have expanded during the last decades in Indonesia. ^1- 5^ Approximately 77.57% of fish produced in freshwater aquaculture in Indonesia are sourced from freshwater ponds and inland waters. ^6^ However, their development depends upon many factors, such as fish species, aquaculture systems, water depletion, fish diseases, farmers’ knowledge, and aquaculture practices. ^4, 7- 10^

Freshwater aquaculture is one of the fastest-growing aquacultures in Indonesia, with more than 3,378,298.92 metric tons produced in 2018. ^6, 11, 12^ Nile tilapia ( *Oreochromis niloticus)* contributed 37.93% of the total aquaculture production, African catfish ( *Clarias gariepinus*) 33.35%, *Pangasius* catfish ( *Pangasius hypophthalmus*) 12.38%, common carp ( *Cyprinus carpio*) 9.28%, and giant gourami ( *Osphronemus goramy*) 6.96%. ^13- 15^

Indonesian giant gourami strains include the local "tambago”, “palapa”, “soang”, “galunggung” and “blusafir" strains, which have been grown semi-intensively in small-scale farms for decades. ^8, 13, 14, 16^ However, they have not been able to contribute majorly to freshwater aquaculture production in Indonesia. Although the gourami sago strain has been approved as a candidate for aquaculture production, its distribution is limited in the West Sumatra Province of Indonesia. ^17, 18^ Therefore the development of its hatchery is necessary to be able to contribute to the production of freshwater aquaculture. The gourami sago strain is considered to support food security along with many other freshwater fish species in Indonesia.

The gourami sago strain was approved as a candidate for freshwater aquaculture in 2018 (Decree of the Ministry of Marine and Fisheries, Republic of Indonesia No.56/KEPMEN-KP/2018). ^19^ However, data on its reproductive characteristics are still limited. The evaluation of reproductive performance in other fish species has had beneficial impacts in the development of freshwater aquaculture in Asia. ^20- 24^ In contrast, there are still gaps in knowledge of giant gourami sago strain broodfish regarding size at oocyte maturity, age of sexual maturity, sperm characteristics, egg hatchability, survival after eyed-egg stage, larva weaning, and growth rate. These factors were identified as key challenges for successful giant gourami sago strain hatchery performance in the future. Therefore, the present study was conducted to evaluate the reproductive characteristics in the giant gourami sago strain to provide basic knowledge for a hatchery development in the future.

## Methods

### Ethical considerations

There are no required permits from the government of the Republic of Indonesia to evaluate reproductive characteristics in gourami sago strain broodfish ( *Osphronemus goramy*) as a candidate for future aquaculture. The study was funded by the Research and Community Service, Universitas Bung Hatta under a competitive grants scheme called the research of Professor in 2021 (Contract number: 06.02.1.46.03.2021). This grant included ethical approval and permits to collect fish specimens, rear and spawn giant gourami sago strains in the Aquaculture Laboratory Faculty of Fisheries and Marine Science, Universitas Bung Hatta facilities. There was no animal suffering involved in this study and gourami sago strain broodfish were still in good condition when returned to the pond. Ethical approval was granted by the Ethics Commission for Research and Community service at Universitas Bung Hatta (023/LPPM/Hatta/I-2021).

### Rearing and selection of breeders

Juvenile fishes were selected about six years ago from a local hatchery in Luhak District, Lima Puluh Kota Regency, West Sumatra Province. The juvenile fish were kept in tanks and transported by truck to the Aquaculture Laboratory, Faculty of Fisheries, and the Marine Science University of Bung Hatta. A total of 200 individuals giant gourami sago strain juvenile fishes were reared for five years until sexual maturity, in an 8.4 m3 (6 × 2 × 0.7 m) concrete freshwater pond. During the rearing of the fishes to sexual maturity, the juvenile fish were fed with commercial fish pellets (781-2 with 30% crude protein content and 4% crude fat; PT Japfa Comfeed Indonesia Tbk).

After the giant gourami sago fish were reared for five years, 20 mature individual broodfish were separated according to sex and reared in two 18 m ^3^ (6 × 2 × 1.5 m) concrete freshwater ponds for 60 days. The broodfish were fed twice daily (09:00 AM and 16:00), with extruded feed pellets containing 39.50% crude protein and 12.21% fat with a predetermined quantity of 3% of fish weight per day. Besides that, the fish were given sente leaves ( *Alocasia macrorriza* L.) at amounts of 1% of fish wet weight per day. The leaves contained 2.85% protein and 0.47% crude fat (of wet weight). Each concrete freshwater pond had a 50 mm drain in the middle, which was covered with a net of 0.5 cm mesh size to prevent the fish from escaping and predators from entering. Water was pumped from borehole wells at a rate of 5 liters per minute.

A total of 20 broodfish with mature oocytes were selected, consisting of 10 females and 10 males. Before spawning, female and male broodfish were weighed using scales (OHAUS model CT 6000-USA with 0.1 g accuracy), and body length was measured using a meter ruler with 1mm accuracy. The average weight and length of the 10 female broodfish were 2140 ± 159 g and 39.70 ± 1.77 cm, while those of the male broodfish were 3060 ± 135 g and 43.1 ± 1.79 cm.

Reproductive parameters in gourami sago strain broodfish were analyzed using the following formulae:

• Condition factor (CF) = wet weight in gram/length ^3^ × 100
• Ovulated egg weight (g) = fish weight before spawning (g) – fish weight after spawning (g)
• Ova somatic index (%) = egg weight ovulation (g)/fish weight before spawning (g) × 100

Absolute fecundity was the total number of eggs estimated per nest, and relative fecundity was the total number of eggs per kg body weight.

### Female reproductive performance

Starting in August 2020 onwards, the broodfish were checked monthly for eggs and semen production. The broodfish were captured with a hand net and anesthetized by oral ingestion of Tricaine methanesulfonate (MS-222, ethyl 4-aminobenzoate methanesulfonate 98%, Sigma Aldrich Co, USA, MO; 50 mg L ^−1^), based on the dosage used for *Hemibagrus wyckii*. ^21^ Oocyte maturation was assessed for each individual. The oocyte maturity in giant gourami females was assessed from oocytes sampled by intraovarian biopsies using a flexible polyethylene catheter. ^21^ Egg diameter was measured using Labo microscope model L-711 and Canon Digital Camera Software 3.

Natural spawning of broodfish was carried out in 1.2 m ^3^ (2 × 1 × 0.6 m) concrete freshwater ponds with a male-female sex ratio of 1:1. Before the broodfish spawned, the ponds were drained, cleaned, and all other species removed. Then, palm fibers were placed on top of a bamboo raft in the pond. The pond was then filled with water, and the female and male broodfish were released into the spawning pond. The male broodfish made a nest for five to seven days, after which spawning took place and the female broodfish laid eggs. Spawning occurred in the afternoon (between 3.00 to 5.00 PM). Due to the presence of a very large oil globule, giant gourami eggs float. ^8^ After the broodfish had finished laying eggs, the eggs were kept by the female broodfish in the nest. After the eggs had been kept by the broodfish for four hours in the nest, the eggs were collected and transferred to an incubation tray, which was placed in a ventricular hatching system. A total of 100 eggs for each broodfish were incubated in incubation trays. Meanwhile, the broodfish were returned to their pond once spawned, and no mortality occurred.

Egg weight and diameter were measured for 25 eggs per female using SHIMADZU-model AY 220 scales with 0.1 mg accuracy and a microscope (Labo model L-711) using Canon Digital Camera Software 3. A total of 25 eggs were randomly sampled 16 hours after spawning to determine the fertility rate (FR). The hatching rate (HR) was determined by counting all hatched fry 48 hours after spawning. Then, the endogenous feeding period of the fish larvae was counted until the egg yolks ran out (in days), and embryo survival rate (%) to the eyed-egg stage was measured.

### Determination of sperm quality

To stimulate the spermiation process in male broodfish, an LHRH-preparation (Ovaprim, manufactured for Syndel Laboratories Ltd, 2595 Mccullough Rd Nanaimo B. C 9VS 4m9 Canada) was injected into male fish, with a dosage of 0.5 ml per kg of the brooder. Semen samples were obtained from 10 gourami sago broodfish randomly selected from the farm. The male broodfish were first anesthetized with 50 mg Lˉ ^1^ of MS-222, ^25^ then fish weights (MaW) and total lengths (MaL) were measured. Special care was taken to avoid any contamination of semen with urine, feces, mucus, and water. Semen samples were collected using plastic syringes in 3 mL aliquots and then placed in an insulated ice-cooled container, transported to the laboratory, and analyzed within two hours.

The sperm assessment included gross (visual) and microscopic examination as reviewed by Rurangwa *et al.* ^26^ and Cabrita *et al.* ^27^ The gross examination was based on visual and physical observation of parameters like semen volume, semen pH, sperm concentration, motility, and duration motility. Semen volume was determined by collecting the semen in a graduated cylinder. Semen pH was determined with a hand pH meter (HI8424 Hanna Instruments, USA). Microscopic examination was carried out using the Olympus model CX40, with × 10 and × 25 magnification, to determine parameters such as motility (MO) percentage and duration, by observing water-activated semen placed on a glass slide. Motile sperm were observed and expressed as a percent of non-moving sperm. Motility duration (DMO) was determined as the period between movements of the sperm to a cessation of any progress using Neubauer’s hemocytometer and calculated as the number of sperm ml ^−1^. ^28^ Semen pH was determined with a hand pH meter (HI8424 Hanna Instruments, USA).

### Water quality

Water samples were collected in the spawning pond and incubation trays to determine alkalinity, hardness, and pH. The protocol for determining alkalinity by standard methodology is presented by Rice *et al.*, 2012. ^29^ pH values were determined with a pH meter (digital mini pH meter, 14pH, IQ Scientific, Chemo-science Thailand Co., Ltd, Thailand). An oxygen meter (YSI model 52, Yellow Spring Instrument Co., Yellow Springs, OH, USA) was used *in situ*, and water temperature in the spawning pond and incubation trays were measured with a thermometer (Celsius scale).

### Statistical analysis

Results were given as the mean values (± SD). Simple linear regression analyses were performed using SPSS software (version 16.0 for Windows; SPSS Inc., Chicago, IL). The standard deviation of each parameter was determined. For linear regression analyses, correlations were considered significant at p < 0.05, and trends or tendencies were considered significant at p < 0.05.

## Results

The reproductive characteristics of female broodfish in sago giant gourami are summarized in Table 1. The total number of eggs per nest (absolute fecundity) varied from 2000 to 2650, while relative fecundity (total number of eggs per kg female brooder) varied between 977 and 1071. The fertility rate ranged from 76 to 84%, and the hatching success rate ranged from 72 to 80%. Endogenous feeding period ranged from 10 to 12 days, and embryo survival rate to eyed-egg stage varied between 94.73 and 95%.

**Table 1.** Reproduction characterization in sago strain of giant gourami females broodfish (Mean ± SD).

	Variables	Range (Min-Max)
Fish length (cm)	39.70 ± 1.77	38˗43
Fish weight before spawning (g)	2140 ± 159.78	1958˗2500	
Fish weight after spawning (g)	2108 ± 157.64	1930˗2465	
Condition factor	3.30 ± 0.42	2.54˗3.86	
Egg weight ovulated (g)	32.80 ± 2.86	28˗38	
Ova somatic index (%)	1.55 ± 0.07	1.43˗1.65	
Absolute fecundity (egg/fish)	2205 ± 201	2000˗2650	
Relative fecundity (egg/kg body weight)	1029 ± 36	977˗1071	
Egg diameter (mm)	2.42 ± 0.05	2.32˗2.46	
Hardened egg diameter (mm)	3.42 ± 0.02	3.40˗3.45	
Egg diameter increase (%)	29.63 ± 1.43	27.8˗32.14	
Egg weight (mg)	10.33 ± 1.09	9.02˗12.20	
Hardened egg weight (mg)	13.36 ± 1.27	11.74˗15.20	
Hardened egg weight increase (%)	22.69 ± 2.24	19.74˗24.81	
Fertility rate (%)	81.60 ± 3.37	76˗84	
Hatching rate (%)	76.40 ± 2.27	72˗80	
Endogenous feeding period *(*day)	11.2 ± 0.63	10˗12	
Embryo survival rate to the eyed-egg stage (%)	94.76 ± 0.42	94.73˗95

Reproductive characteristics for male broodfish and sperm samples are presented in Table 2. The average live weight of the males was 3340 ± 275.68 g. Sago giant gourami male broodfish were found to be slightly bigger than female broodfish. Gonad weight ranged from 25 to 30 g, whereas gonad somatic index ranged from 0.83 to 0.93%.

**Table 2.** Reproduction characterization in sago strain of giant gourami males broodfish (Mean ± SD).

	Variables	Range (Min-Max)
Fish weight (g)	3060 ± 134.99	2800˗3200
Fish length (cm)	43.1 ± 1.79	40˗ 5	
Condition factor	3.74 ± 0.43	3.08˗4.38	
Gonads weight (g)	27.5 ± 1.72	25˗30	
Gonadosomatic index (%)	0.90 ± 0.03	0.83˗0.94	
Semen volume (mL per kg body weight)	0.60 ± 0.12	0.4˗0.7	
Semen pH	8.18 ± 0.15	7.9˗8.4	
Sperm concentration (10 ^9^/mL)	1.44 ± 0.14	1.2˗1.6	
Motility (%)	70.04 ± 2.27	68˗75	
Duration of motility (sec)	50.2 ± 7.25	43˗61

The linear correlation ( *r* ^2^) between variables of reproduction characterization parameters in sago strain of giant gourami females broodfish results are shown in Table 3. In this study, the reproductive parameters that showed strong correlations with the absolute fecundity were female fish weight before spawning ( *r* ^2^ = 0.921) and female fish weight after spawning ( *r* ^2^ = 0.864). Similarly, results revealed significant correlations between egg diameter and hardened egg diameter ( *r* ^2^ = 0.833), and between egg diameter and percentage of the hardened diameter ( *r* ^2^ = 0.699). Meanwhile, egg diameter and fertility rate were moderately correlated ( *r* ^2^ = 0.568). In contrast, the egg diameter was not strongly related to absolute fecundity ( *r* ^2^ = 0.169) and relative fecundity ( *r* ^2^ = 0.096). On the other hand, the survival rate of larvae (10 days) also had strong correlations with the hatching rate ( *r* ^2^ = 0.998) and endogenous feeding period ( *r* ^2^ = 0.757).

**Table 3.** Correlations of variables ( *r* ^2^) in sago strain of giant gourami females broodfish.

	FEL	FWBS	FWAS	CF	OEW	OVI	AF	RF	EW	HEW	EWI	ED	HED	HDI	FR	HR	EFP
FEL																	
FWBS	**0.720**																
FWAS	**0.717**	**0.999**															
CF	**0.757**	**0.575**	**0.574**														
OEW	**0.539**	**0.565**	**0.553**	0.365													
OVI	0.281	0.191	0.012	0.000	**0.774**												
AF	0.255	**0.921**	**0.864**	**0.637**	0.387	0.072											
RF	0.012	0.207	0.063	0.011	0.065	0.321	**0.524**										
EW	**0.894**	**0.659**	**0.655**	**0.677**	0.552	0.246	**0.567**	0.041									
HEW	**0.841**	**0.631**	**0.626**	**0.514**	**0.582**	0.295	0.468	0.004	**0.924**								
EWI	0.165	0.109	0.109	0.354	0.033	0.000	0.010	0.002	**0.924**	0.041							
ED	0.164	0.132	0.338	0.131	0.378	**0.688**	0.169	0.096	0.030	0.263	0.000						
HED	0.064	0.029	0.237	0.126	0.025	0.207	0.022	0.064	0.468	0.184	0.030	**0.833**					
HDI	0.266	0.293	0.342	0.209	0.103	0.085	0.373	0.195	0.318	0.298	0.006	**0.699**	0.294				
FR	0.026	0.229	0.020	0.000	0.135	0.264	0.000	0.004	0.067	0.004	0.160	**0.568**	0.064	0.054			
HR	0.035	0.020	0.060	0.046	0.000	0.009	0.003	0.009	0.226	0.108	0.364	0.018	0.001	0.143	**0.703**		
EFP	0.113	0.186	0.190	0.094	0.006	0.098	0.231	0.103	**0.747**	**0.806**	0.147	0.317	0.001	0.116	0.015	0.013	
SR	0.070	0.032	0.034	0.027	0.007	0.003	0.112	0.024	0.194	0.019	0.005	0.033	0.000	0.324	0.063	**0.998**	**0.757**

Statistically important at *r* ^2^ > 0.500 (underlined).

Abbreviations: FeL: female fish length (cm); FWBS: female fish weight before spawning (g); FWAS: female fish weight after spawning (g); CF: condition factor; OEW: egg weight ovulation (g), OVI: Ova somatic index (%), AF: absolute fecundity (eggs), RF: relative fecundity (eggs), EW: egg weight (mg), HEW: Hardened egg weight (mg), EWI: eggs weight increase (%), ED: egg diameter (mm), HED: hardened egg diameter (mm), HDI: hardened egg diameter increase (%), FR: fertility rate (%), HR: hatching rate (%), EFP: endogenous feeding period (days), SR: survival rate (10 days).

Linear correlation analysis results ( *r* ^2^) between variables of reproduction characterization parameters in sago giant gourami male broodfish are shown in Table 4. The reproductive parameters that had a strong correlation with gonad weight were somatic index of gonads ( *r* ^2^ = 0.836), while semen volume ( *r* ^2^ = 0.521), semen pH ( *r* ^2^ = 0.521) and sperm concentration ( *r* ^2^ = 0.506) were moderately correlated with gonad weight. In contrast, the gonadal weight negatively correlated with sperm motility ( *r* ^2^ = 0.017) and duration of motility ( *r* ^2^ = 0.275). In addition, the sperm concentration was also moderately correlated with the sperm motility ( *r* ^2^= 0.556) and duration of motility ( *r* ^2^= 0.502).

**Table 4.** Correlations of variables ( *r* ^2^) in sago strain of giant gourami males broodfish.

	MaL	MaW	CF	GW	GI	SV	pH	SC	MO
MaL	-								
MaW	**0.714**								
CF	**0.807**	0.347							
GW	0.399	**0.550**	0.187						
GI	0.003	0.042	0.071	**0.836**					
SV	0.025	**0.576**	0.042	**0.521**	0.000				
pH	**0.516**	**0.772**	0.353	**0.521**	0.127	0.296			
SC	0.186	**0.661**	0.131	**0.506**	0.068	0.425	**0.645**		
MO	0.068	0.453	0.061	0.017	0.130	0.393	0.280	**0.556**	
DMO	0.159	0.322	0.083	0.275	0.012	0.082	0.430	**0.502**	**0.519**

Statistically important at *r* ^2^ > 0.500 (underlined).

Abbreviations: MaW: male fish weight (g), MaL: male fish length (g), CF: condition factor; GW: gonadal weight (g); GI: gonad somatic index (%), SV: semen volume (ml), SC: sperm concentration (10 ^9^/mL), MO: motility (%), DMO: duration of motility (sec).

The physico-chemical water quality parameters in the spawning ponds and incubation trays for embryo development were as follows: water alkalinity ranged from 50.5 mg L ^−1^ to 52.5 mg L ^−1^ HCO _3_^−^, hardness varied from 65.5 mg L ^−1^ to 67.5 mg L ^−1^ CaCO _3_, pH ranged from 6.4 to 6.6, oxygen ranged from 6.1 mg L ^−1^ to 6.7 mg L ^−1^, and temperature varied from 28°C to 30°C.

## Discussion

In our study, body weight in female sago giant gourami broodfish before spawning ranged from 1958 to 2500 g per fish, and ova somatic index ranged from 1.43 to 1.65%. Body weight in female sago strain gourami broodfish was smaller than that of giant gourami belonging to the galunggung strain, which ranged from 2500 to 3500 g. ^8^ Conversely, the ova somatic index of galunggung strain is found to be slightly bigger than that of sago giant gourami, which ranged from 3.7 to 4.6%. ^8^ The differences in reproductive characteristics in broodfish can be explained by strains, brood size, age of broodfish, previous spawning history, and the production setting. ^23^ Absolute fecundity in sago giant gourami ranged from 2000 to 2650 eggs fish ^−1^ and relative fecundity (RF) ranged from 977 to 1071 eggs kg ^−1^. Egg produced in kg fish ^−1^ (RF) is thought to be more informative than absolute fecundity. RF in sago strain of giant gourami was smaller compared to those in galunggung strain, palapah strain, and blusafir strain. ^8, 16, 31^ On the other hand, the difference in relative fecundity can also be related to differences in broodfish size and age used. ^23^ Environmental factors such as rainfall also influenced the number of eggs per spawning in giant gourami broodfish, while the water temperature negatively related to the number of eggs per spawning. ^15^ Furthermore, egg diameter in sago strain of giant gourami was found to be almost the same as other strains of giant gourami ( Table 5). In this study, average egg diameter was 2.42 ± 0.05 mm, consistent with those reported by other researchers; 2.18 ± 0.19 mm for the giant gourami, ^30^ 2.40 ± 0.05 mm for blusafir strain, ^16^ and 2.5 ± 0.05 mm for galunggung strain. ^8^ The differences in the RF, ova somatic index, egg diameter, and hatching rate of giant gourami can be influenced by differences in the strains. Furthermore, egg diameter has been influenced by dietary protein level, ^32- 35^ age of broodfish, ^36^ and spawning season. ^37, 38^ In our study, egg diameter was shown to be positively correlated with egg weight, hardened egg weight, and egg weight increase. The egg weight of rainbow trout also increased after the hardening process and was positively correlated with the viability of eggs. ^39^ Other egg quality metrics, such as hatching rate, and survival to first feeding, have been correlated with good egg quality. ^21^

**Table 5.** Summary of the fecundity, gonadal somatic index, egg diameter, and hatching rate of giant gourami.

Species	Strain	Relative fecundity (egg/kg fish)	GSI (%)	Eggs diameter (mm)	Hatching rate (%)	Reference
*Osphronemus goramy*	Sago	1037 ± 90	1.91 ± 0.35	2.42 ± 0.05	76.40 ± 6.33	This study
*Osphronemus goramy*	Bastar	2423 ± 348	2.78 ± 1.16	2.2 ± 0.2	96.36 ± 2.30	16
*Osphronemus goramy*	Galunggung	4011 ± 287	4.15 ± 0.63	2.5 ± 0.05	89.3 ± 1.30	8
*Osphronemus goramy*	-	5508 ± 1547	2.32 ± 0.50	2.18 ± 0.19	61.60 ± 0.0	30
*Osphronemus goramy*	Tambago	2.896 ± 185	3.16 ± 0.11	2.47 ± 0.03	91.06 ± 4.06	31

In this study, the hatching rate of the embryo in the sago strain of giant gourami was smaller than those of other strains of giant gourami. ^8, 16, 31^ This condition might be affected by the egg and sperm quality in giant gourami sago strain broodfish. In the present study, whether eggs and sperm quality of sago giant gourami breeders are affected by feed type was poorly understood. Broodfish sex ratio did not influence egg quality. ^8^ The reproductive parameters that were strongly correlated with the hatching rate were fertility rate ( *r* ^2^ = 0.703) and survival rate (10 days) ( *r* ^2^ = 0.998). According to Sink *et al*., ^40^ the biochemical composition of broodfish eggs is strongly correlated with egg quality. In this study, we did not evaluate the biochemical composition of an egg, because the relationship between egg quality and biochemical composition is difficult to interpret. ^41^

The keys regulator hormones of fish reproduction are gonadotropins, follicle-stimulating hormone, luteinizing hormone, and sex steroids. ^42, 43^ In addition, oocyte development and maturation are also regulated by locally acting paracrine and autocrine signaling. ^44, 45^ However, there is no information about the effects of such factors on oocyte development in giant gourami sago. Still, the extrusion feed enriched with vitamin E (d-α-tocopherol) at concentrations of 137.8, 238.05, 338.72 and 439.39 mg per kg feed affected markers of reproductive functions of giant gourami broodfish, such as the sexual maturity cycles, ovum somatic index, relative fecundity, and egg diameter. ^31^

Various efforts have been made by scientists to increase the reproductive performance of female broodfish, such as increasing dietary protein levels for *Xiphophorus helleri*, ^33^ *Channa marulius*, ^46^ and *Ictalurus punctatus*. ^40^ Additionally, implantation of 17ß-estradiol has also improved the reproductive performance in *Hemibagrus nemurus*. ^47^ Currently, whether the increase in the protein level of feed and use of hormones can increase the reproductive potential in sago giant gourami is poorly understood. Therefore, we recommend the use of proteins in feed (at levels of 25%, 30%, or 35%) and the addition of 17β-estradiol (for example 200, 400, or 600 μg/kg body weight) to increase the reproductive potential of giant gourami sago in the future.

Average semen volume in giant gourami sago was lower (0.4 to 0.6 ml) than those of *Hemibagrus wyckii* (0.60 to 1.20 ml), ^21^ but higher than those of *Pterygoplichthys gibbiceps*. ^48^ It appears that the semen volume depends on fish species. ^21, 49, 50^ Many factors influenced sperm quality and quantity such as genetic, physiological, spawning season, and environmental factors. ^26, 49, 51, 52^ On the other hand, improvements in feed nutrition of broodfish can increase gamete quality and semen volume. ^46^ Commercial honey combined with 10% Dimethyl Sulfoxide (DMSO) was also shown to increase sperm motility. ^53^ Synthetic hormones such as gonadotropin-releasing hormone analogs (GnRHa), with or without dopamine antagonist, and domperidone (Dom) effectively improve sperm quality. ^54, 55^ Nevertheless, the duration of motility of sperm was strongly correlated with the water quality in ponds. In this study, the water quality parameters in spawning ponds included alkalinity of 50.5 to 52.5 mg/L HCO _3_^−^ and hardness of 65.5 to 67.5 mg/L CaCO _3_, a pH between 6.4 and 6.6, and a water temperature between 28 and 30 ^o^C. These water quality parameters were able to support the ability of the sperm to fertilize the egg. ^21^

The sperm motility in sago giant gourami ranged from 68 to 75%, and the duration of motility ranged from 43 to 61 sec. These results are consistent with *Genypterus blacodes* and *Esox lucius*. ^50, 56^ Sperm motility includes the percentage of motile sperm, straight-line velocity, curvilinear velocity, average path velocity, and linearity. ^50^ In this study, we did not investigate those parameters. In addition, the percentage of motile sperm is influenced by the addition of extenders and cryoprotectants. ^57- 59^ However, sperm motility from fresh semen was slightly greater compared to cryopreserved semen from *Esox lucius*. ^50^ The fertility rate of eggs ranged from 76 and 84%; however, no significant correlation was detected between fertility rate and sperm parameters, such as semen volume, semen pH, motility, and duration of motility. Conversely, the sperm concentration was moderately correlated with sperm motility and duration of motility. The parameters commonly measured to assess sperm quality in brood were volume, density, and motility (such as the percentage of motile sperm, straight-line velocity curvilinear velocity, average path velocity, linearity, and amplitude of lateral head displacement), including fertilizing capacity. ^49, 50, 52, 60^ In this study, we did not investigate the ionic composition of the semen, but this phenomenon could be related to the ionic composition of semen which might have a significant influence on sperm motility and duration of motility.

## Conclusions

This research analyzed the reproductive characteristics of giant gourami sago strain broodfish reared in concrete freshwater ponds, in the Aquaculture Laboratory Faculty of Fisheries and Marine Science, Universitas Bung Hatta. Relative fecundities of the giant gourami sago strain broodfish ranged from 977 to 1071 eggs, and egg diameter ranged from 2.32 to 2.46 mm. Semen volume ranged from 0.4 to 0.7 ml per kg body weight and sperm motility was comprised between 68 and 75%. A strong linear relationship was observed between absolute fecundity and female fish weight before and after spawning. Similarly, a strong, positive correlation was observed between survival rate (10 days) and hatching rate. The sperm concentration was also moderately positively correlated with the motility and duration of sperm motility. Keys to increasing the reproduction performance in gourami sago strain fish depend on broodfish weight, relative fecundity, and hatching rates. Although data on the reproductive characteristics of gourami sago strain broodfish have been obtained, there are still knowledge gaps in feeding technologies and larval weaning during rearing. Therefore, for successful practices in hatcheries, further research is recommended to determine a proper feed formulation and the development of appropriate aquaculture systems.

## Data availability

### Underlying data

Figshare: Reproduction characterization of the gurami sago ( *Osphronemus goramy* Lacepède, 1801): basic knowledge for a hatchery development strategy for the future.

https://doi.org/10.6084/m9.figshare.14661189.v3. ^61^

This project contains the following underlying data:

- Table 1. Raw data of fish length, weight, absolute fecundity, and relative fecundity of gurami sago broodfish
- Table 2. Raw data of egg diameter (mm) in sago strain of giant gourami broodfish
- Table 3. Raw data of hardened egg diameter (mm) in sago strain of giant gourami broodfish
- Table 4. Raw data of egg diameter increase (%) in sago strain of giant gourami broodfish
- Table 5. Raw data of egg weight (mg) in sago strain of giant gourami broodfish
- Table 6. Raw data of hardened egg weight (mg) in sago strain of giant gourami broodfish
- Table 7. The data of egg weight increase (%) in sago strain of giant gourami broodfish
- Table 8. The data of fertilization rate (%) in sago strain of giant gourami broodfish
- Table 9. The data of hatching rate (%), endogenous feeding period (day), and embryo survival rate to the eyed-egg stage (%) and in sago strain gourami
- Table 10. Male size, gonadal weight, and semen in sago strain of giant gourami broodfish
- Table 11. Sperm concentration (10 ^9^/mL) in sago strain of giant gourami broodfish
- Table 12. Sperm Motility (%) in sago strain of giant gourami broodfish
- Table 13. Duration motility (sec) in sago strain of giant gourami broodfish

Data are available under the terms of the Creative Commons Attribution 4.0 International license (CC-BY 4.0).
