# Supplementary material for: Reproductive characteristics of the giant gurami sago strain ( Osphronemus goramy Lacepède, 1801): basic knowledge for a future hatchery development strategy
Source: F1000Res. 2022 Apr 25;10:922. Originally published 2021 Sep 15. [Version 2] doi: 10.12688/f1000research.53760.2 (PMC9127375; doi:10.12688/f1000research.53760.2)
Supplement: Peer review history

# Peer review: Reviewer response for version 2

**DOI:** 10.5256/f1000research.133416.r135817

**Published:** 2022-05-23

**Authors:** Mochamad Syaifudin

**Affiliations:** 1 Faculty of Agriculture, Program Study of Aquaculture, Universitas Sriwijaya, Prabumulih, Indonesia

The article explaining reproduction performances of the giant gurami sago strain was well written and recommended to index.

Is the work clearly and accurately presented and does it cite the current literature?

Yes

If applicable, is the statistical analysis and its interpretation appropriate?

Yes

Are all the source data underlying the results available to ensure full reproducibility?

Yes

Is the study design appropriate and is the work technically sound?

Partly

Are the conclusions drawn adequately supported by the results?

Yes

Are sufficient details of methods and analysis provided to allow replication by others?

Yes

Reviewer Expertise:

Fish genetics and reproduction

I confirm that I have read this submission and believe that I have an appropriate level of expertise to confirm that it is of an acceptable scientific standard.

# Peer review: Reviewer response for version 1

**DOI:** 10.5256/f1000research.57175.r129705

**Published:** 2022-04-19

**Authors:** Mochamad Syaifudin

**Affiliations:** 1 Faculty of Agriculture, Program Study of Aquaculture, Universitas Sriwijaya, Prabumulih, Indonesia

In general, the article is quite good explaining the reproductive performances of gourami sago. However, the introduction needs to state the distribution of gourami strain sago in Indonesia, whether it is endemic or introduced in the region. There is less information on the general characteristics of sago compared to other strains. Table 5 should explain some specific conditions of breeding technology used in other strains.

Is the work clearly and accurately presented and does it cite the current literature?

Yes

If applicable, is the statistical analysis and its interpretation appropriate?

Yes

Are all the source data underlying the results available to ensure full reproducibility?

Yes

Is the study design appropriate and is the work technically sound?

Partly

Are the conclusions drawn adequately supported by the results?

Yes

Are sufficient details of methods and analysis provided to allow replication by others?

Yes

Reviewer Expertise:

Fish genetics and reproduction

I confirm that I have read this submission and believe that I have an appropriate level of expertise to confirm that it is of an acceptable scientific standard, however I have significant reservations, as outlined above.

# Peer review: Reviewer response for version 1

**DOI:** 10.5256/f1000research.57175.r129704

**Published:** 2022-04-06

**Authors:** Fatimah Hashim

**Affiliations:** 1 Faculty of Science and Marine Environment, Universiti Malaysia Terengganu, Kuala Terengganu, Malaysia

I have evaluated this article and found the description of the approach taken for this study to be very appropriate, and each description is very detailed. Comparisons between previous studies are also fully informed in this article. I strongly support the following step of indexing of this article for the sharing of information on the reproductive characteristics of the giant gurami sago. I only found that a few corrections to the abstract need to be made, wherein the method section and the brand name need to be deleted from this section.

Is the work clearly and accurately presented and does it cite the current literature?

Yes

If applicable, is the statistical analysis and its interpretation appropriate?

Yes

Are all the source data underlying the results available to ensure full reproducibility?

Yes

Is the study design appropriate and is the work technically sound?

Yes

Are the conclusions drawn adequately supported by the results?

Yes

Are sufficient details of methods and analysis provided to allow replication by others?

Yes

Reviewer Expertise:

Fish amoeba, protozoology, cell biology

I confirm that I have read this submission and believe that I have an appropriate level of expertise to confirm that it is of an acceptable scientific standard.
